# Tetrahedral honeycomb surface reconstructions of quartz, cristobalite and stishovite

**DOI:** 10.1038/s41598-018-29853-1

**Published:** 2018-08-09

**Authors:** Oleg D. Feya, Qinggao Wang, Sergey V. Lepeshkin, Vladimir S. Baturin, Yurii A. Uspenskii, Artem R. Oganov

**Affiliations:** 1 Moscow Institute of Physics and Technology, Dolgoprudny, Moscow Region 141700 Russia; 2 Skolkovo Institute of Science and Technology, Skolkovo Innovation Center, Nobel St. 3, Moscow, 143026 Russia; 3 P.N. Lebedev Physical Institute, Russian Academy of Sciences -, 119991 Leninskii Ave. 53, Moscow, Russia; 4 International Center for Materials Discovery, Northwestern Polytechnical University, Xi’an, 710072 China; 5 School of Physics and Electronics, Henan University, Kaifeng, 475004 China

## Abstract

Crystalline silica (SiO_2_) is a major material used in many technologies, yet the exact surface structures of silica polymorphs are still mostly unknown. Here we perform a comprehensive study of surface reconstructions of α-cristobalite (001), α-quartz (001) and stishovite (110) and (100) using evolutionary algorithm USPEX in conjunction with *ab initio* calculations. We found the well-known “dense surface” to be among low-energy reconstructions of α-quartz (001), as well as its previously proposed distorted version, which we call “shifted surface”. For cristobalite and stishovite we show the formation of reconstructions without dangling bonds which share common features with well-known “dense surface” of α-quartz (001). We call them “dense cristobalite” and “dense stishovite” – all of these have honeycomb arrangements of corner-sharing SiO_4_-tetrahedra in the surface layers. These tetrahedral honeycombs have very low surface energies, and such tetrahedral surface pattern is observed even in stishovite (the bulk structure of which has SiO_6_-octahedra, rather than SiO_4_-tetrahedra).

## Introduction

Silica is used in many technologies as a ceramic, glass, catalyst, abrasive, low-k material in microelectronics, piezoelectric material, optical fiber material, etc^1^. Silica surfaces play an important role in interactions with water, proteins, and other organic molecules.

Silica has many crystalline polymorphs, e.g. α- and β-quartz, α- and β-cristobalite, tridymite, coesite, stishovite, and others^2^. Crystal structures of most silica polymorphs are composed of corner-sharing SiO_4_-tetrahedra, arranged in very diverse topologies^3,4^. Low-temperature polymorphs such as α-quartz and α-cristobalite^5,6^ can exist at ambient conditions. Stishovite, a high-pressure phase composed of SiO_6_-octahedra, is metastable at normal conditions^7^. While bulk structures and properties of silica polymorphs are well known, rather few experimental studies explored the surface structure of silica.

There are several theoretical studies of surface reconstructions of α-quartz^4,8–11^, and a few experiments^12,13^. The (001) surface of α-quartz is a peculiar case: never present in natural crystals, it is only seen in synthetic crystals of quartz, yet usually not as a flat surface, but a complex pattern of pyramids^14,15^. This is the most studied of silica surfaces. Low-energy electron diffraction showed that α-quartz (001) surface has (1 × 1) pattern^16^, but later work reported huge $(\sqrt{84}\times \sqrt{84})$ R11° reconstruction existing above 600 °C^12^. In theoretical works “dense surface” with cell (1 × 1) was considered to be the stable reconstruction of α-quartz (001)^8^. Some studies show two reconstructions, (2 × 1) and (2 × 2), comparable in energy^17,18^.

α-cristobalite (space group *P*4_1_2_1_2) is a well-known metastable phase at ambient conditions, but the structure of its surfaces is still unknown^5^. Likewise, little is known about stishovite sufraces: there  is only one article describing surface properties of cleaved (110) face^19^. Stishovite (100) surface does not reconstruct at 0 K, but has reconstructions above 40 K described as collective atomic motion^20^.

Given rather detailed information about the (001) surface of α-quartz (001), we use it as a test system to show the power of our methodology for surface structure prediction, and then predict essentially unknown stable reconstructions of α-cristobalite (001) and stishovite (110), (100) surfaces. As we will see, the former two reconstructions share some similarities with “dense surfaces” mentioned above – they have no dangling bonds and can be described as a honeycomb made of corner-sharing SiO_4_-tetrahedra. So we call these reconstructions “dense cristobalite” and “dense stishovite”.

## Computational Methodology

We used the USPEX code^21–24^ for evolutionary structure prediction. The technique implemented in this code allows one to automatically explore low-energy configurations and find stable and many low-energy metastable surface reconstructions, with variable number of both surface atoms and surface unit cells, covering all physically allowed values of chemical potentials. The search was performed within a set of supercells (including 1 × 1, 1 × 2, 2 × 1, 1 × 3, 3 × 1, 2 × 2, 1 × 4 and 4 × 1), allowing the algorithm to add on top of the substrate up to 2 Si and 4 O atoms per unit cell, and up to 3 Si and 6 O in case of quartz (001). During the evolutionary search the first generation (containing 80 structures) was produced by random structure generator. Each of the subsequent generations consisted of 50 structures, obtained by applying 30% heredity, 20% softmutation, 10% transmutation and 40% random structure generator. Each structure was carefully relaxed in five stages, starting from low and ending with high precision. When the set of stable structures remained unchanged for 10 consequent generations, the calculation stopped.

Structure relaxations within the evolutionary search were done using density functional theory (DFT)^25,26^ within the Perdew-Burke-Ernzerhof (PBE) generalized gradient approximation (GGA)^27^ and projector-augmented wave (PAW)^28,29^ method, as implemented in the VASP code^30–32^. The plane wave kinetic energy cutoff was set to 400 eV, and uniform Г-centered k-meshes with reciprocal-space resolution of 2π × 0.07 Å^−1^ were used for Brillouin zone sampling. Only atoms in the surface layer and uppermost 3 Å of the substrate (the “buffer” layer) were allowed to relax, while the other atoms remained fixed in the bulk configuration to simulate the semi-infinite crystal. For the lowest-energy structures we used symmetric surfaces to carefully calculate their energies and avoid dipole fields and the influence of dangling bonds at the bottom of the surface. To cancel interactions between slabs and their periodic images we have adopted dipole corrections^33^.

For evolutionary search we used the slab models of four O-Si-O trilayers for α-cristobalite and stishovite, and three trilayers for α-quartz. The thickness of the vacuum layer in the initial stages of relaxation was set to 11 Å, then increased to 12 Å and finally to 13 Å in the end of relaxation. Low-energy structures were used for further analysis, we added up to four trilayers per cell (1 × 1) to the bottom of the slabs, used 13 Å vacuum layers and fixed only bottom layers to recalculate the energies more precisely. We found no significant differences comparing to results of relaxation with 3 Å thick buffer layer.

To examine stability of surfaces we adopted previously described method^24,34^. The stability of structures is determined by the surface energy:

1 $${\rm{\Delta }}{G}_{sur}=1/N[{G}_{sur}-\sum {n}_{i}{\mu }_{i}]$$

where *G*_*sur*_ is the Gibbs free energy of a candidate structure, *n*_*i*_ and *μ*_*i*_ are the numbers of atoms and chemical potential of i-th species, respectively. *N* equals to (m*n) for a (*m*x*n*) surface cell, and serves for normalization of Gibbs free energies.

*μ*_*O*_ and *μ*_*Si*_ should satisfy the following three conditions: (a) ${\mu }_{O}\le 1/2{\mu }_{{O}_{2}}$, (b) ${\mu }_{Si}\le {\mu }_{Si}^{bulk}$ and (c) ${\mu }_{Si}+2{\mu }_{O}={G}_{Si{O}_{2}}$. Given much greater entropy of the gas phase compared to solid phases, Gibbs free energies of solids can be replaced by internal energies of structures at 0 K^35^ We can rewrite (1) as

2 $${\rm{\Delta }}{G}_{sur}=1/N[{E}_{sur}-{n}_{Si}{E}_{Si{O}_{2}}-{\mu }_{O}({n}_{O}-2{n}_{Si})]$$

where ${E}_{Si{O}_{2}}$ and *E*_*sur*_ are the total energy (per unit cell) of the corresponding bulk structure (cristobalite, quartz and stishovite) and the candidate surface model, respectively.

${\mu }_{Si}$-independent (*E*_0_) and stoichiometry deviation (Δ*n*) terms are defined as

3 $$\begin{array}{cc}({\rm{a}}) & {E}_{0}=1/N[{E}_{sur}-{n}_{{\rm{S}}{\rm{i}}}{E}_{Si{O}_{2}}],\\ (b) & {{\rm{\Delta }}}_{n}=1/N({n}_{o}-2{n}_{si})\end{array}$$

respectively. As a result we plot *E*_0_ (Δ*n*) graphs for each surface, where each reconstruction is represented by a point, and stable structures form a convex hull, while metastable ones are above the convex hull.

Surface energy was calculated as $\gamma =({E}_{sur}-{E}_{Si{O}_{2}})/(2A)$, where *E*_*sur*_ and ${E}_{Si{O}_{2}}$ are the energies of the slab and of the bulk SiO_2_, containing the same number of formula units as the slab; *A* is the surface area of one side of the slab. The factor “2” takes into account that each slab has two surfaces.

## Results

Cristobalite at high temperatures crystallizes in the cubic form (*Fd-*3*m*), and is stable between 1743 K and 2000 K. At lower temperatures, when cristobalite is metastable and temperature is dropped below 548 K, symmetry is lowered to *P*4_1_2_1_2, and α-cristobalite is formed^36^.

**Figure 1:**
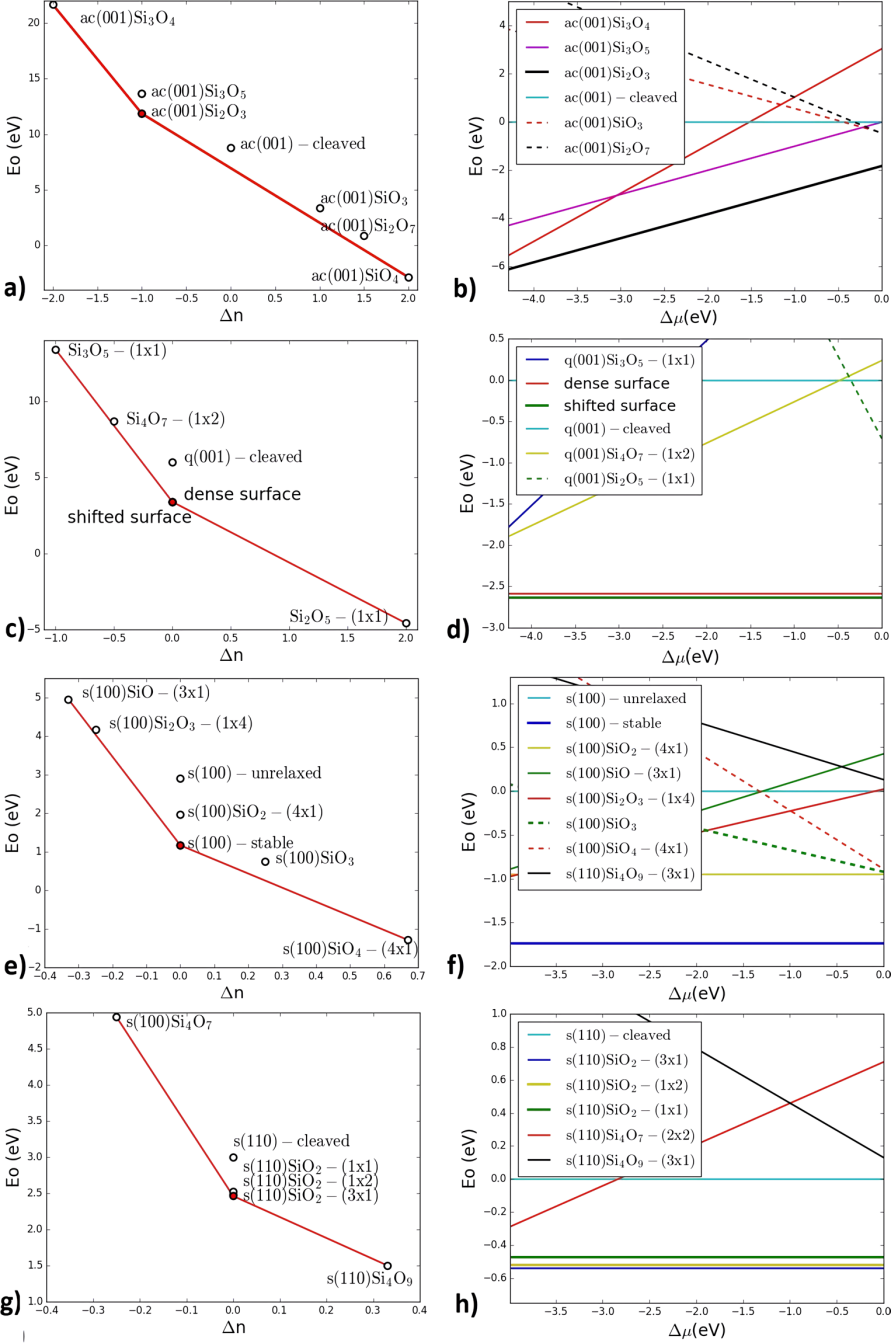
Stability regions of different silica surface reconstructions plotted as a function of stoichiometry deviation Δn (left) and of oxygen chemical potential Δμ_o_ (right). (**a**,**b**) α-cristobalite (001); (**c**,**d**) α-quartz (001); (**e**,**f**) stishovite (110); (**g**,**h**) stishovite (001). In all of these cases, each surface has only one stable structure in the entire range of allowed chemical potentials.

For α-cristobalite we have found one stable reconstruction ac(001)Si_2_O_3_-(1 × 2) (Fig. 1a,b) (‘ac’ stands for α-cristobalite). We call it “dense cristobalite” as it shares features common with well-known quartz (001) “dense surface”^8^. We also observed two peculiar metastable reconstructions containing reactive oxygen radicals: ac(001)Si_3_O_5_-(1 × 1) and ac(001)Si_3_O_4_-(1 × 1), as they lie only ~0.04 eV/atom above convex hull.

**Figure 2:**
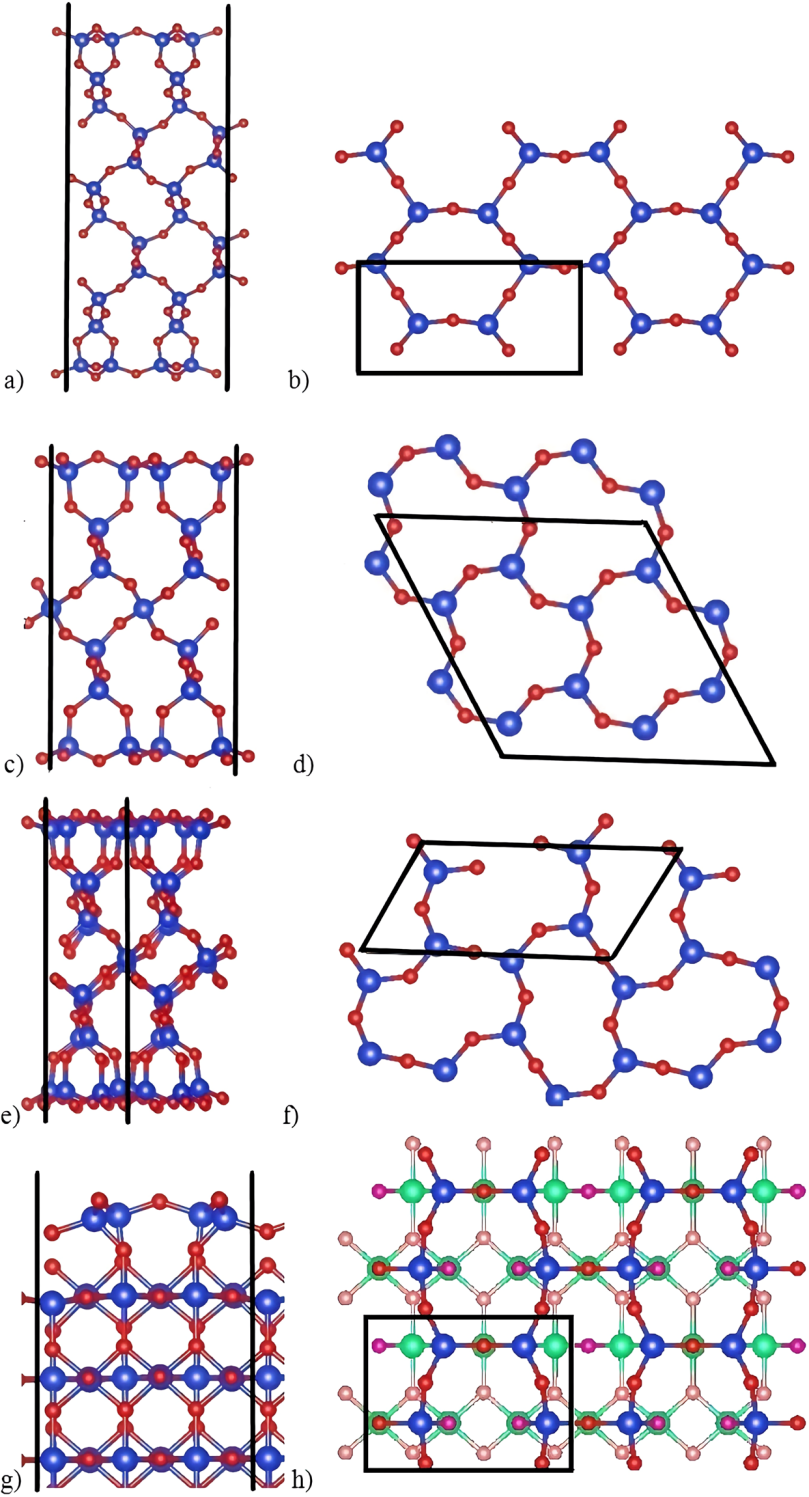
“Dense” surfaces of silica polymorphs: (**a**,**b**) “Dense cristobalite”, side and top views; (**c**,**d**) “Dense surface” of quartz (001), side and top view; (**e**,**f**) “Shifted surface” of quartz (001), side and top views; (**g**,**h**) “Dense stishovite”, side and top views. Blue and red balls are silicon and oxygen atoms. Purple atoms are subsurface oxygens in stishovite. Each surface has four-coordinate Si and two-coordinate O-atoms on the top, and the surface layer can be described as a honeycomb made of corner-sharing SiO_4_-tetrahedra.

According to our calculations, α-quartz (001) has two low-energy reconstructions (Fig. 1c,d). One of them is the above mentioned “dense surface”. The other is q(001)SiO_2_-(1 × 2) (‘q’ stands for quartz), which we call «shifted surface» (Fig. 1c,d). In the direction of *a-*vector it is similar to “dense surface”, but in the *b-*direction it looks like “optimized surface”, explored by Malyi *et al*.^18^ (Fig. 2c–f).

We also considered stishovite (100) and (110) surfaces. As mentioned above, according to the theoretical study^20^ stishovite (100) surface does not reconstruct at zero temperature. Our results confirm only one stable structure for stishovite (100) surface, which is the same as relaxed cleaved surface (Fig. 1e,f). Stable reconstruction for stishovite (110) s(110)SiO_2_-(3 × 1) we call “dense stishovite” (Fig. 1g,h).

To calculate energies of these reconstructions with higher accuracy, we have created symmetric surfaces. For quartz such a model consists of 36 formula units, both reconstructions were expanded to 2 × 2 supercells to compare two surfaces with the same number of atoms. In previous works^18^ this number of trilayers was shown to be appropriate for precise calculations. The same energy was obtained after increasing the slab up to 45 formula units. For “dense cristobalite” we used symmetric surface with 28 formula units. We compared it with 32-layer symmetric surface, which had energy lower by 0.0015 eV/atom, a negligible difference, which justified the use of a smaller slab to reduce computational costs. For the non-trivial “dense stishovite” surface we used a huge 38-layer model. Substrate of stable reconstruction of stishovite (100) was made of 39 trilayers.

**Figure 3:**
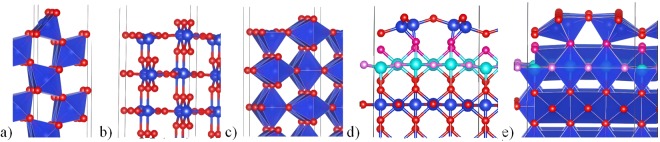
Stishovite (100) and (110) surfaces: (**a**) stable cleaved (100) surface; (**b**,**c**) unstable cleaved (110) surface; (**d**,**e**) stable “dense stishovite” (110) reconstruction. Ball-and-stick and polyhedral representations are given. Blue and red balls are silicon and oxygen atoms. Purple atoms are interlayer oxygens. It is quite surprising that cleaved surface, without any reconstruction, is stable for stishovite (100) surface. The (110) surface of stishovite reconstructs dramatically, and with the bulk structure recovering only in the third polyhedral layer; the second layer being similar to the first layer of the cleaved (110) surface.

Unreconstructed cleaved surfaces have dangling bonds and are highly unstable. Cleaved O-terminated slab of cristobalite after relaxation has surface energy 2.830 J/m^2^, while transformation into the “dense” reconstruction lowers it to 0.560 J/m^2^ (Table 1) The same takes place  in quartz. O-terminated slab has surface energy 2.183 J/m^2^ (Si-termination is even more unstable), but “dense surface” is much more stable, its energy being 0.413 J/m^2^, which agrees with previous result^18^ of 0.400 J/m^2^. “Shifted surface” is slightly more stable with the surface energy of 0.404 J/m^2^. Unrelaxed slab of stishovite (100) has surface energy 2.09 J/m^2^, while after relaxation it drops down to 0.839 J/m^2^ (Fig. 3a). Surprisingly, the evolutionary search provided this structure to be the most stable reconstruction for stishovite (100) surface. As a check, we performed two evolutionary searches, differing in the termination of the substrate (one with siloxane bridges on the top and Si dangling bond, and the other with oxygen dangling bond), and got the same resulting reconstruction. For stishovite (110) we used the same cleaved surface as Muscenti *et al*.^19^ did (Fig. 3b). Our calculated surface energy is 1.03 J/m^2^, quite similar to 1.13 J/m^2 ^^37^ of their paper. Stable reconstruction has surface energy of mere 0.447 J/m^2^, as a result of absence of dangling bonds on “dense” surfaces.

**Table 1:** Surface energies and band gaps for considered “dense” surfaces and unreconstructed surfaces of α-cristobalite, α-quartz and stishovite.

Surface	Surface energy, J/m^2^	Band gap, eV
Bulk α-cristobalite		5.55
O-terminated α-cristobalite (001)	2.830	2.41
Dense cristobalite (001)	0.560	5.06
Bulk α-quartz		5.74
O-terminated α-quartz (001)	2.183	3.55
Dense surface (001)	0.413	5.64
Shifted surface (001)	0.404	5.52
Bulk stishovite		5.45
Stable stishovite (100)	0.839	4.53
Cleaved stishovite (110)	1.030	3.83
Dense stishovite (110)	0.447	3.86

Uppermost regions of all of these surfaces contain oxygen atoms, followed by Si atoms slightly below (Fig. 2). They form siloxane bridges. These surfaces share similar “honeycomb” topology despite very different substrates to which they are attached. This can be explained by the well-known high flexibility of Si-O-Si angles between the tetrahedra. The [SiO_4_] tetrahedron is very rigid and its deformations (e.g., changing O-Si-O angles from 109.5°) require very high energy, but it takes only 6.7 kJ/mol to change Si-O-Si angle from 130° to 180°^3,37^. This is why “honeycomb” surface, being so flexible, matches so easily such different substrates as α-quartz, α-cristobalite and stishovite.

“Dense cristobalite” has two types of siloxane bridges (Fig. 2a,b). One has Si-O bonds of 1.67 Å length and Si-O-Si angle 138.2°. Another bridge is almost straight – with Si-O-Si angle 173.9° and Si-O bond lengths at 1.64 Å. The 3-membered silicon rings (3MR) normal to surface are slightly deformed.

Si-O-Si bridges of “dense surfaces” of quartz (Fig. 2c,d) have angles of 117.9°, which is consistent with previous studies^8,18^. Bond lengths are 1.615–1.627 Å, close to values in the bulk (1.626 Å), but the angles differ from those in crystals (which are equal to ~143°). A more stable quartz reconstruction is called “shifted surface” (Fig. 2e,f) because 3MR are shifted in opposite directions along *b* axis. This surface was theoretically predicted in 2008^17^ and confirmed experimentally in 2015^13^. Here this surface reconstruction was obtained automatically, which shows the power of our approach.

“Dense stishovite” has two types of Si-O-Si bridges (Fig. 2g,h). Bridges on the short side of six-membered rings (6MR) are concave and directed into the bulk. Their angles are equal to 133.3°, and Si-O bonds are 1.65 Å. Convex bridges have Si-O-Si angles 144.4° and Si-O bond lengths 1.62 Å. Looking along the *a*-direction, these two bridges form a wavy pattern going through surface.

Gao *et al*.^38^ described a few examples of 2D-silica. δ-2D-silica with *P* $\bar{4}\,$*m*2 symmetry (stoichiometry SiO_2_) was built on the basis of “dense surface”. One can also find elements of “dense surface” in γ-2D-silica (*P* $\bar{4}\,$*m*2), yet siloxane Si-O-Si angles there are 113°. α-2D-silica with symmetry group *P*6/*mmm* consists of 2 layers with Si_2_O_3_ stoichiometry (the same as for “dense cristobalite”), connected via Si-O-Si bonds normal to the surface. Comparing the “dense surface” of quartz (001) and its close relative, the “shifted surface”, we see that the latter is symmetry-broken, with elliptically distorted 6MR.

On the cleaved stishovite (100) surface, the SiO_6_-octahedron becomes a square pyramid with Si dangling bond (Fig. 3a). After relaxation its volume slightly increases – by 1.7%, while subsurface octahedra below become slightly smaller. Top siloxane bridge gets a wider angle – 103.8° against 99.5° for unrelaxed surface. This is accompanied by shortening top Si-O bonds from 1.74 Å to 1.69 Å.

“Dense stishovite” (110) reconstruction is beautiful and has a remarkably low surface energy, so let us consider it in more detail. While all Si atoms in bulk stishovite are 6-coordinate, surface Si atoms reduce their coordination number to 4, which helps to stabilize the surface, because SiO_4_-tetrahedra are the building blocks of silica at normal conditions. Unreconstructed surface (Fig. 3b,c) has 5-coordinate Si atom with one dangling bond. Its surface consists of 2 polyhedra, one is the same SiO_6_-octahedron as in the bulk, the other is a SiO_5_-square pyramid. The “dense stishovite” surface contains two geometrically inequivalent SiO_4_-tetrahedra (Fig. 3e), with volumes 2.21 Å^3^ and 2.38 Å^3^, which is very similar to the SiO_4_-tetrahedra in quartz (2.20 Å^3^), and with bonds lengths around 1.62 Å.

The SiO_4_-tetrahedra of the “dense stishovite” surface (and all the other “dense surfaces” discussed here) form a distorted honeycomb that shares strong similarities with Si-O tetrahedral layers of sheet silicates such as micas, kaolinite Al_4_[Si_4_O_10_](OH)_8_^39^ and serpentine (Mg,Fe)_3_Si_2_O_5_(OH)_4_^40^.

Top layers of “dense stishovite” reconstruction are shown in Figs 2h and 3d,e. First and second layers are connected via siloxane bridges with angles 136.8°.

**Figure 4:**
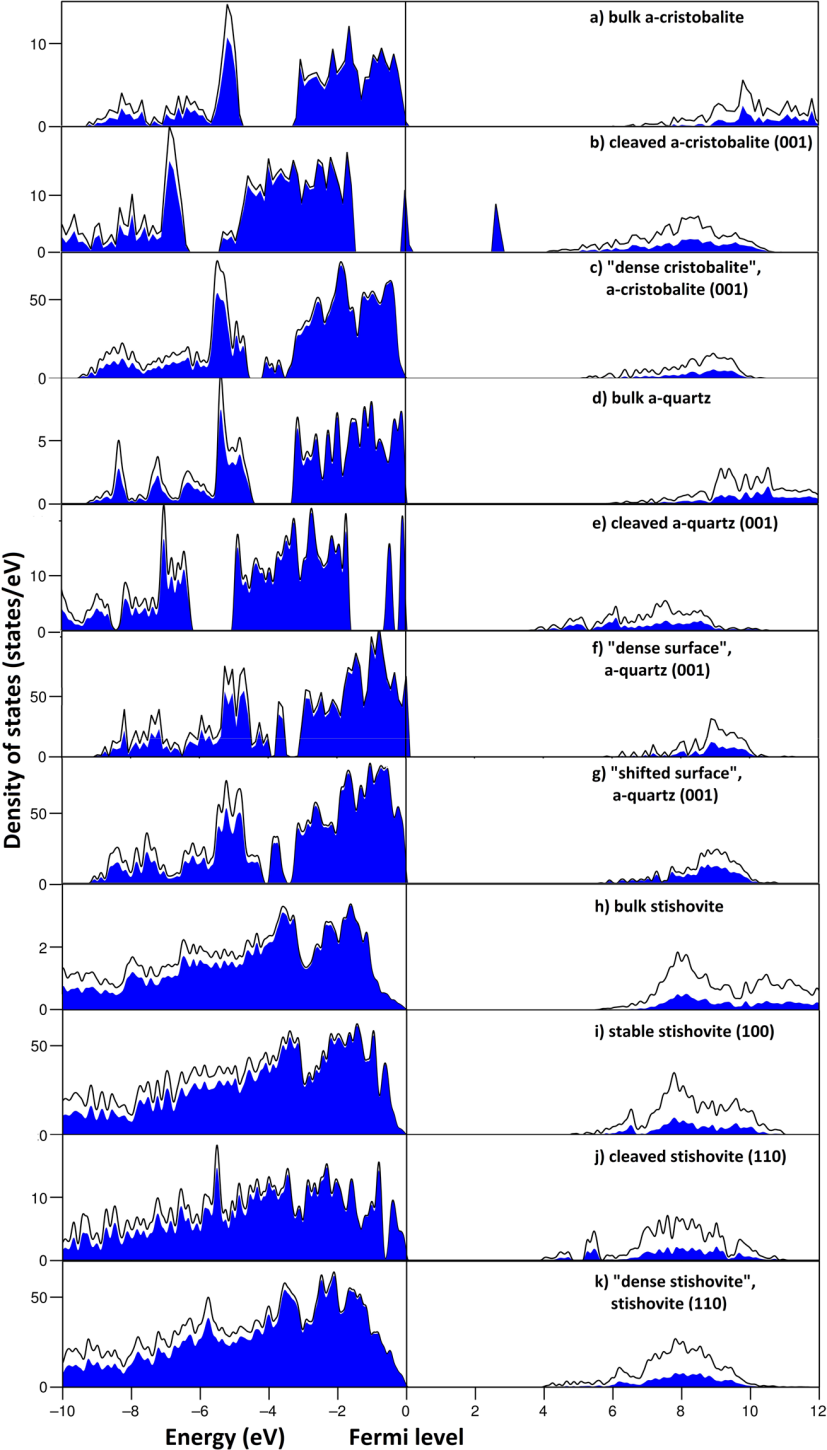
DFT densities of states of α-cristobalite (001), α-quartz (001), stishovite (100) and (110), and bulk silica structures. Black lines represent total DOS, while O-p states, which make the main contribution to valence states, are highlighted by blue color. Cleaved surfaces of cristobalite and quartz possess O-p peaks at the Fermi level, while “dense surfaces” of all mentioned polymorphs have electronic structures close to corresponding bulk structures.

We have calculated electronic properties for cleaved and “dense” surfaces (Fig. 4). Dangling bonds of a cleaved α-cristobalite produce two high peaks of the electronic density of states, dominated by O-p, one half-filled peak at Fermi level and the other unoccupied (Fig. 4b). These peaks reduce the DFT band gap from 5.55 eV for bulk structure (Fig. 4a) to just 2.41 eV. In contrast, “dense cristobalite” has no surface states in the band gap, and its band gap equal to 5.06 eV (Fig. 4c). Its DOS is almost identical to bulk, except for lower conduction band minimum (CBM).

Peaks near valence band maximum (VBM) of cleaved quartz (001) also originate from O-p states of dangling bonds, but are more localized than those in cristobalite (Fig. 4c). Band gap reduces from 5.74 eV (for the bulk, Fig. 4d) to 3.55 eV, which agrees with previous studies^8,18^. “Dense surface” and “shifted surface” have almost identical electronic properties, except differences in band gap (5.64 eV and 5.52 eV for “dense surface” and “shifted surface”, respectively) (Fig. 4f,g), which is consistent with Malyi *et al*.^18^, where slightly smaller (by 0.08 eV) band gaps were reported.

Electronic properties of cleaved stishovite (110) surface (Fig. 4j) were described by Muscenti *et al*.^19^. O-p states near VBM split because of very different environments of the oxygen atom at the surface and in the bulk. Band gap reduces from 5.45 eV in the bulk to 3.83 eV (3.60 eV in previous work^19^). “Dense stishovite” DOS is similar to the DOS of the bulk (Fig. 4k). It has the band gap of 3.86 eV, slightly higher than that of the cleaved surface.

While due to the dangling bonds all cleaved surfaces tend to split O-p states near band gap, or create peaks in the band gap, “dense” surfaces’ have no dangling bonds and their electronic DOSs are similar to bulk DOS with no presence of electronic  states between valence and conduction bands and rather wide band gaps.

From the viewpoints of crystal chemistry and electronic structure it is easy to understand the stability of “dense surfaces”. The formation of “honeycomb” reconstructions eliminated dangling bonds. This, in turn, affects the electronic structure: while for cleaved surfaces we observe surface states within the gap, these vanish in the case of “dense surfaces”, resulting in wider gaps and electronic stabilization of surface reconstructions^41^.

## Conclusions

We have performed a systematic evolutionary search for stable reconstructions of several silica surfaces: α-cristobalite (001), α-quartz (001) and stishovite (001) and (110). According to our calculations, each of these surfaces has only one stable reconstruction. In case of quartz two reconstructions are close in energy. We see that many low-index  surfaces of crystalline silica polymorphs tend to create stable reconstructions in the form of “dense” surfaces. “Dense cristobalite”, “dense surface” of α-quartz, “dense stishovite” share some similarities: they have the same structural topology, have no dangling bonds, and surprisingly even for stishovite (the bulk structure of which is based on SiO_6_-octahedra) the “dense” surface reconstruction is based on SiO_4_-tetrahedra. From theoretical calculations all of these surfaces have rather wide band gaps (the DFT band gaps range from ~4 eV for “dense stishovite” to 5–6 eV for cristobalite and quartz), and their DOSs are close to bulk, implying chemical inertness of these surfaces. Quartz and cristobalite dust are known as carcinogens, in contrast to stishovite^42^. One hypothesis connects toxicity of silica dust with the presence of reactive oxygen species on the surface, and these were found with electron paramagnetic resonance in cristobalite dust^43^ and theoretically predicted on α-quartz (011) surface^44^ and in silica nanoclusters^45^. “Dense” surfaces described here have no reactive oxygen species. In a follow-up paper we will examine other surfaces of silica polymorphs, to check whether they have reactive oxygen species.

## Electronic supplementary material

Supplementary Information
