# Supplementary material for: Tetrahedral honeycomb surface reconstructions of quartz, cristobalite and stishovite
Source: Sci Rep. 2018 Aug 9;8:11947. doi: 10.1038/s41598-018-29853-1 (PMC6085356; doi:10.1038/s41598-018-29853-1)
Supplement: Supplementary file 1 — Supplementary Information [file 41598_2018_29853_MOESM1_ESM.pdf]

Tetrahedral honeycomb surface reconstructions of quartz, cristobalite and stishovite

Oleg D.Feya[1*], Qinggao Wang[5], Sergey V.Lepeshkin[2,3], Vladimir S.Baturin[2,3] , Yurii A. Uspenskii[3] and Artem R. Oganov[2,1,4]*

[1] Moscow Institute of Physics and Technology, Dolgoprudny, Moscow Region 141700, Russia
[2] Skolkovo Institute of Science and Technology, Skolkovo Innovation Center, Nobel St. 3, Moscow 143026, Russia.
[3] P.N. Lebedev Physical Institute, Russian Academy of Sciences - 119991 Leninskii Ave. 53, Moscow, Russia
[4] International Center for Materials Design, Northwestern Polytechnical University, Xi'an 710072, China
[5] School of Physics and Electronics, Henan University, Kaifeng 475004, China

* Correspondence to feyaolkodmi@gmail.com and a.oganov@skoltech.ru

Supplementary information

1. Initial substrates for evolutionary search

Structure	Composition (Si-O)	Cell shape (a,b,c in Å)	Cell shape (α,β,γ)
O-terminated α-quartz (001)	3 - 6	4.91, 4.91, 4.89	90°, 90°, 120°
O-terminated α-cristobalite (001)	4 - 6	4.98, 4.98, 6.45	90°, 90°, 90°
Si-terminated stishovite (100)	4 - 6	4.18, 2.67, 8.35	90°, 90°, 90°
O-terminated stishovite (110)	4 - 6	2.67, 5.91, 5.24	90°, 90°, 90°

Here, *c* means the thickness of the substrate in the direction perpendicular to the slab

2. POSCAR files of discussed surfaces

Dense cristobalite

```
1.00000000000000
   9.9559999999999995   0.0000000000000000   0.0000000000000000
   0.0000000000000000   4.9779999999999998   0.0000000000000000
   0.0000000000000000   0.0000000000000000  41.3988000000000014
Si O
28 56
Direct
 0.1613091805894626  0.2188405858909590  0.3703307939771889
 0.6612512718107766  0.2343835090263653  0.3693379400635024
 0.1946694945627669  0.7187382922252894  0.3265755575132090
 0.6971414103956022  0.7383365256453800  0.3257391559042233
 0.4433803734795205  0.7664996022601471  0.2824910021939004
```

0.9468812196948093	0.5718134309986311	0.2826586133309661
0.4394974460421324	0.2676954555589504	0.2390537195792746
0.9419582899688166	0.0716695821793465	0.2388442121185648
0.0900566221762773	0.9802951977359839	0.6051142356134704
0.5772398271830426	0.4999648389997375	0.0898618965477098
0.5898179173298104	0.4301044410620705	0.6054103973382894
0.0772268398979605	0.9476087476365933	0.0898658239459209
0.2766897166282689	0.4390848035730528	0.6054619810753366
0.7638598220566095	0.9587281894687081	0.0896273833923829
0.7765009794120772	0.9713922567640623	0.6052634903227272
0.2638641439901903	0.4888820713283764	0.0897240561837620
0.1993584099971315	0.7040051258815083	0.4999392494807395
0.6900074703304924	0.2210435252363681	0.1951369405761199
0.4138265403482038	0.0651406336120317	0.4119451744192943
0.1849932789349609	0.2096724754644725	0.5434748767286450
0.6721312081586959	0.7282796625094150	0.1515608851993164
0.4489438374365449	0.5624907897808740	0.4552144008442340
0.6953596500015848	0.7097329877983398	0.5000345492117475
0.1901235335000280	0.2300497130732921	0.1950162868940168
0.9101985987674510	0.2545646430684911	0.4139908750617067
0.6815522745078155	0.1991373493337321	0.5435373816206877
0.1729878604186865	0.7228068253395676	0.1515680081029060
0.9452148754119918	0.7548738657751670	0.4571608832806859
0.1848396067814662	0.7117158425311274	0.6076304682661089
0.6722625631184656	0.2316933508898771	0.0874605855046795
0.6823258952443041	0.1989608750795782	0.6237885722162773
0.1695087960500388	0.7168566617458225	0.0713370848481958
0.6847766146685359	0.6986530454159663	0.6077170561236969
0.1720549644097176	0.2162456052260708	0.0875081320616431
0.1819972300954831	0.2110159952044341	0.6237179959916404
0.6691016084861019	0.7311624760474942	0.0712787490314142
0.4335597455375364	0.4714854983842400	0.6190791316646127
0.9211001998011241	0.9902144502011723	0.0761931123650470
0.9336881930865815	0.9386290550204777	0.6186093895868368
0.4210493973310392	0.4567922813285392	0.0762731720388672
0.1317882985663061	0.4551661441846377	0.5204229335519983
0.6203473304930753	0.9722100671049105	0.1750479499628170
0.4478684996395117	0.2526864556685453	0.4429791724531995
0.2957567520003366	0.3449484430720986	0.5677092721493651
0.7827518499714330	0.8650622118126634	0.1274044664437000
0.3339703721248100	0.5949688233204071	0.4825019658749454
0.8270441907729946	0.1160055915400733	0.2117994264409901
0.2436939033025425	0.9622601645183195	0.5218997189128274
0.7305500150174211	0.4792714287761086	0.1728467175673885
0.4234287040293694	0.7542419312805393	0.4239010698700696
0.0924711898852593	0.8048450956741391	0.4728826340335885
0.5859925669831014	0.3191493526362805	0.2229496057732518
0.0714016985502397	0.0775317027624922	0.5673346541433091
0.5583287810246915	0.5983858591553997	0.1275997554298556
0.7367388241171113	0.4446582832043120	0.5211765509701962
0.2321559264423030	0.9755717609816301	0.1721256774878270
0.9304174153459570	0.4439517428035202	0.4456028863895938
0.7944611318197232	0.0661905273334398	0.5674576757934489
0.2827569084123454	0.5828691931934387	0.1274830794146666
0.8326661529166728	0.8276242877066622	0.4842338216962645

```
0.3258005705309728  0.3406173870450928  0.2123178072422576
0.6258789699537317  0.9518360016079299  0.5211680976164246
0.1208978412807724  0.4822500406975507  0.1755084909462354
0.9266196126928250  0.9439045635449475  0.4254575939822445
0.5938113828359946  0.6239609974362637  0.4712636648870401
0.0873396967213864  0.1236769732457077  0.2224840306563536
0.5699595716465709  0.3328758177889597  0.5677205390675581
0.0582405020810270  0.8492576885512406  0.1275971173519252
0.2630001750359270  0.1218893959294221  0.3987410878919775
0.7609914185574880  0.3067844878526316  0.3992664725247366
0.0215402893052001  0.3220208473466641  0.3864182271497967
0.5191863528402436  0.1210323954976289  0.3828228204275916
0.2277913401393477  0.4694812940694106  0.3508035449885156
0.6318714437171593  0.5035231405718363  0.3481990224329365
0.1277634853727037  0.9686644356123537  0.3462179684732689
0.7307661191556249  0.0035998286857151  0.3471295563955223
0.3335723253964247  0.8275842771886559  0.3105704966913194
0.8365830590619439  0.6334027304802134  0.3103099980168125
0.0951623324654420  0.6144222723170003  0.2981206245901618
0.5928493587422281  0.8181792004069592  0.2970270368183137
0.4256362209534288  0.4573273775533764  0.2707752591331172
0.9260521063123051  0.2638099363351856  0.2704922786362189
0.4219348190862817  0.9588013533614230  0.2511331020452729
0.9305206752854573  0.7625877112885604  0.2510529074554810
```

Dense surface, α-quartz (001)

```
1.00000000000000
     9.8260002136230469    0.0000000000000000    0.0000000000000000
    -4.9130001068115234    8.5095663070678711    0.0000000000000000
     0.0000000000000000    0.0000000000000000   32.7028007507324219
Si O
36 72
Direct
0.2470896418506072  0.2469729961354545  0.2944204633681764
0.2637552302837634  0.0074667135386406  0.2368698565797160
0.4211023803476195  0.3144308998666361  0.5450962036049276
0.0937025846573647  0.1462154284354540  0.5444279332955162
0.2612540437250246  0.2615775955003627  0.4650548292034742
0.0081786469899399  0.4820392685375054  0.3510525093887793
0.2734273459157919  0.0160465670643077  0.4077009417161577
0.1047970498283064  0.4024776943039328  0.1565889340703421
0.4320481306198900  0.0612622776140412  0.1574090123695839
0.7470896269494460  0.2469729961354545  0.2944204633681764
0.7637552302837705  0.0074667135386406  0.2368698565797160
0.9211023803476195  0.3144308998666361  0.5450962036049276
0.5937026070091065  0.1462154284354540  0.5444279332955162
0.7612539841203798  0.2615775955003627  0.4650548292034742
0.5081786665477139  0.4820392685375054  0.3510525093887793
0.7734273459157919  0.0160465670643077  0.4077009417161577
0.6047970349271452  0.4024776943039328  0.1565889340703421
0.9320481008175676  0.0612622776140412  0.1574090123695839
0.2470896716529296  0.7469730408389381  0.2944204633681764
0.2637552600860857  0.5074667824565111  0.2368698565797160
0.4211023803476195  0.8144308998666361  0.5450962036049276
0.0937026070091065  0.6462154433366152  0.5444279332955162
0.2612540735273470  0.7615776551050075  0.4650548292034742
0.0081786930904073  0.9820393877467950  0.3510525093887793
0.2734273459157919  0.5160465735835658  0.4077009417161577
```

0.1047970796306288	0.9024777539085775	0.1565889340703421
0.4320481306198900	0.5612623074163636	0.1574090123695839
0.7470896865540908	0.7469730408389381	0.2944204633681764
0.7637552302837705	0.5074667824565111	0.2368698565797160
0.9211023803476195	0.8144308998666361	0.5450962036049276
0.5937026070091065	0.6462154433366152	0.5444279332955162
0.7612540437250246	0.7615776551050075	0.4650548292034742
0.5081787261523587	0.9820393877467950	0.3510525093887793
0.7734273459157919	0.5160465735835658	0.4077009417161577
0.6047970945317900	0.9024777539085775	0.1565889340703421
0.9320481604222124	0.5612623074163636	0.1574090123695839
0.4486885918476915	0.1705229943774924	0.5575978736374338
0.0632217713051375	0.4788053534400660	0.5619881283985322
0.2557606054234185	0.2825998019895621	0.5648278550831876
0.4134960858322998	0.3268926945841812	0.4952379919658512
0.2338087628517940	0.3987347672418764	0.4463910013520476
0.3793153468172221	0.3165292358892557	0.3303839107217144
0.4339453432186957	0.0458187391906506	0.3855459013661502
0.1359031432159625	0.4471319595596412	0.3731281707608858
0.1101570180245375	0.1521467178045199	0.4942676117848919
0.2899640386500408	0.1768777799798329	0.4261298985956685
0.0839525408732271	0.1142267552999741	0.3150999337455787
0.2316231716083195	0.3906435615351995	0.2751889461228458
0.2936330619485830	0.1714491122686326	0.2565738203715711
0.1121406514820862	0.4234587662818896	0.2064065813051172
0.4149705038550024	0.0499730242474783	0.2075766566817450
0.0771628100858734	0.2307476877623955	0.1443420164976956
0.2702613546152079	0.0356607680897127	0.1368480492794291
0.4627843041215982	0.4247263987266265	0.1396487016049974
0.9486885918476915	0.1705229943774924	0.5575978736374338
0.5632217750304278	0.4788053534400660	0.5619881283985322
0.7557606203245868	0.2825998019895621	0.5648278550831876
0.9134960858322998	0.3268926945841812	0.4952379919658512
0.7338087777529552	0.3987347672418764	0.4463910013520476
0.8793153468172221	0.3165292358892557	0.3303839107217144
0.9339453730210181	0.0458187391906506	0.3855459013661502
0.6359031730182849	0.4471319595596412	0.3731281707608858
0.6101570403762793	0.1521467178045199	0.4942676117848919
0.7899640088477184	0.1768777799798329	0.4261298985956685
0.5839525408732271	0.1142267552999741	0.3150999337455787
0.7316232312129642	0.3906435615351995	0.2751889461228458
0.7936330619485830	0.1714491122686326	0.2565738203715711
0.6121406365809250	0.4234587662818896	0.2064065813051172
0.9149704442503506	0.0499730242474783	0.2075766566817450
0.5771627951847123	0.2307476877623955	0.1443420164976956
0.7702613546152079	0.0356607680897127	0.1368480492794291
0.9627842743192758	0.4247263987266265	0.1396487016049974
0.4486885918476915	0.6705230539821372	0.5575978736374338
0.0632217750304278	0.9788053534400660	0.5619881283985322
0.2557606352257409	0.7825998913965293	0.5648278550831876
0.4134961156346222	0.8268927839911484	0.4952379919658512
0.2338087777529552	0.8987348268465212	0.4463910013520476
0.3793153468172221	0.8165292656915781	0.3303839107217144
0.4339453432186957	0.5458187801688439	0.3855459013661502
0.1359031879194461	0.9471319893619636	0.3731281707608858
0.1101570254751181	0.6521467625080035	0.4942676117848919
0.2899640386500408	0.6768777799798329	0.4261298985956685
0.0839525557743883	0.6142267702011353	0.3150999337455787
0.2316231865094807	0.8906435615351995	0.2751889461228458
0.2936330619485830	0.6714491867744385	0.2565738203715711
0.1121406663832474	0.9234588258865344	0.2064065813051172
0.4149705336573248	0.5499730689509690	0.2075766566817450
0.0771628249870346	0.7307477175647179	0.1443420164976956

0.2702613844175303 0.5356608202437769 0.1368480492794291
0.4627843041215982 0.9247264285289489 0.1396487016049974
0.9486886514523363 0.6705230539821372 0.5575978736374338
0.5632217750304278 0.9788053534400660 0.5619881283985322
0.7557606203245868 0.7825998913965293 0.5648278550831876
0.9134960858322998 0.8268927839911484 0.4952379919658512
0.7338087777529552 0.8987348268465212 0.4463910013520476
0.8793153468172221 0.8165292656915781 0.3303839107217144
0.9339453730210181 0.5458187801688439 0.3855459013661502
0.6359031730182849 0.9471319893619636 0.3731281707608858
0.6101570403762793 0.6521467625080035 0.4942676117848919
0.7899640088477184 0.6768777799798329 0.4261298985956685
0.5839525408732271 0.6142267702011353 0.3150999337455787
0.7316231716083195 0.8906435615351995 0.2751889461228458
0.7936331215532277 0.6714491867744385 0.2565738203715711
0.6121406961855698 0.9234588258865344 0.2064065813051172
0.9149705634596401 0.5499730689509690 0.2075766566817450
0.5771628547893570 0.7307477175647179 0.1443420164976956
0.7702614142198527 0.5356608202437769 0.1368480492794291
0.9627843339239206 0.9247264285289489 0.1396487016049974

Shifted surface, α-quartz (001)

1.00000000000000
9.8260002136230469 0.0000000000000000 0.0000000000000000
-4.9130001068115234 8.5095663070678711 0.0000000000000000
0.0000000000000000 0.0000000000000000 38.2974014282226563
Si O
36 72
Direct
0.2262130943202507 0.2219364318211916 0.3755403707956333
0.2220995696963044 0.7095002116278124 0.3663061481017422
0.2601059166297262 0.4690096686228031 0.3226347004805064
0.1991281248193673 0.9670208517161569 0.3212735442750940
0.2707174059439410 0.2088400178021104 0.5159450669652585
0.2006781444716452 0.7113411605862865 0.5169467256402811
0.4946220775260031 0.4507354263609002 0.4216778905594794
0.4907048739910636 0.9468088638125067 0.4160176530508721
0.2548424619196652 0.4701218590027239 0.4714094371320527
0.2446127090219434 0.9552349872979207 0.4636194648433758
0.4543672962972707 0.2839456279688619 0.5830490982457377
0.4507478799880857 0.5310651343885056 0.2578291803124486
0.3481490125905466 0.7924092591625609 0.5857761530930290
0.3470093859270591 0.0317885119481431 0.2509995891320855
0.1251876219108183 0.1082803490942226 0.5845258397648934
0.1197350566622788 0.3585569056236011 0.2542324450088742
0.0186868716465725 0.6126475706825119 0.5841630677065623
0.0187511397582512 0.8703300098186944 0.2542567277856875
0.7262130943202507 0.2219364318211916 0.3755403707956333
0.7220995845974656 0.7095002116278124 0.3663061481017422
0.7601059166297262 0.4690096686228031 0.3226347004805064
0.6991281546216896 0.9670208517161569 0.3212735442750940
0.7707174357462634 0.2088400178021104 0.5159450669652585
0.7006781593727993 0.7113411605862865 0.5169467256402811
0.9946220477236807 0.4507354263609002 0.4216778905594794
0.9907048739910636 0.9468088638125067 0.4160176530508721
0.7548424619196652 0.4701218590027239 0.4714094371320527
0.7446127388242658 0.9552349872979207 0.4636194648433758
0.9543673260995931 0.2839456279688619 0.5830490982457377
0.9507478501857634 0.5310651343885056 0.2578291803124486
0.8481490423928761 0.7924092591625609 0.5857761530930290
0.8470094157293815 0.0317885119481431 0.2509995891320855

0.6251875921084959	0.1082803490942226	0.5845258397648934
0.6197350715634400	0.3585569056236011	0.2542324450088742
0.5186868809597982	0.6126475706825119	0.5841630677065623
0.5187511174065094	0.8703300098186944	0.2542567277856875
0.2319159206201817	0.3387062174131685	0.5000847588524238
0.1555902282490536	0.8159486871944424	0.4920121592961806
0.3666630111481766	0.2831354126659207	0.4042755667209477
0.3783012361540585	0.8014492439097154	0.3906518141263362
0.4143017711854284	0.5193361628667645	0.4488426872944515
0.4025488930466565	0.9568683426781419	0.4508203164813196
0.1123232242820293	0.4095710368362120	0.4431822501946598
0.1349145991258638	0.9231015238307236	0.4293213748086728
0.1278303851910749	0.0904634877265025	0.5418633718182804
0.1175467718392227	0.3477945572558667	0.2969996837912632
0.0463535561761006	0.6095029520563031	0.5417928652595734
0.0406433701322584	0.8967604065678856	0.2968710737827180
0.2921395494612611	0.1205426229884381	0.4825031915066376
0.2579903473084002	0.6136557334308321	0.4929442109615039
0.1792660163145712	0.7513628567201991	0.6023299866816814
0.1810502607202693	0.0024126719181581	0.2355028502139476
0.3751584566830175	0.6437331313977026	0.5926845973936636
0.3764925513217179	0.8900958615970893	0.2388697844161243
0.2930578184410422	0.1424926612339164	0.6002849564338533
0.2895179622519350	0.3947572452077637	0.2395138850936362
0.4828832616712546	0.4462765998289697	0.6003663463689719
0.4874006004030988	0.6993133984426478	0.2433267314258671
0.4880390143712177	0.9511097468682550	0.6027550237195811
0.4853658363668814	0.1985983677964143	0.2368592142913712
0.4291825089052139	0.2837909211331819	0.5404681797215929
0.4230627656565886	0.5204504450257517	0.3006010247601338
0.3440808984602057	0.8192267371142847	0.5436886595687724
0.3440536594151169	0.0268723327964295	0.2937126684052132
0.0999273778645602	0.2573892073937785	0.5925844077596594
0.0939931642316907	0.5062027620447083	0.2453892199223802
0.2402858720463925	0.1228873362545144	0.3434994732897110
0.2546924403327466	0.6259421802717640	0.3331709367254163
0.2390831424047164	0.3803609317867966	0.3594613315782098
0.1781986163698193	0.8336776213852559	0.3491267247364505
0.0585921710493693	0.1117535948575537	0.3947061510630334
0.0816933219682596	0.5817426118472113	0.3911057142087202
0.7319158908178593	0.3387062174131685	0.5000847588524238
0.6555902580513759	0.8159486871944424	0.4920121592961806
0.8666630111481766	0.2831354126659207	0.4042755667209477
0.8783012361540585	0.8014492439097154	0.3906518141263362
0.9143018009877508	0.5193361628667645	0.4488426872944515
0.9025488930466636	0.9568683426781419	0.4508203164813196
0.6123232242820293	0.4095710368362120	0.4431822501946598
0.6349146214776056	0.9231015238307236	0.4293213748086728
0.6278304298945585	0.0904634877265025	0.5418633718182804
0.6175467345863197	0.3477945572558667	0.2969996837912632
0.5463535710772547	0.6095029520563031	0.5417928652595734
0.5406433775828390	0.8967604065678856	0.2968710737827180
0.7921395792635835	0.1205426229884381	0.4825031915066376
0.7579903771107226	0.6136557334308321	0.4929442109615039
0.6792660163145712	0.7513628567201991	0.6023299866816814
0.6810502756214305	0.0024126719181581	0.2355028502139476
0.8751584864853399	0.6437331313977026	0.5926845973936636
0.8764925811240403	0.8900958615970893	0.2388697844161243
0.7930577886387198	0.1424926612339164	0.6002849564338533
0.7895179622519350	0.3947572452077637	0.2395138850936362
0.9828832318689322	0.4462765998289697	0.6003663463689719
0.9874006302054212	0.6993133984426478	0.2433267314258671
0.9880390143712106	0.9511097468682550	0.6027550237195811

0.9853658065645590 0.1985983677964143 0.2368592142913712
0.9291825387075363 0.2837909211331819 0.5404681797215929
0.9230627656565886 0.5204504450257517 0.3006010247601338
0.8440808984602057 0.8192267371142847 0.5436886595687724
0.8440537190197617 0.0268723327964295 0.2937126684052132
0.5999274225680438 0.2573892073937785 0.5925844077596594
0.5939931418799560 0.5062027620447083 0.2453892199223802
0.7402858720463925 0.1228873362545144 0.3434994732897110
0.7546924701350690 0.6259421802717640 0.3331709367254163
0.7390831722070388 0.3803609317867966 0.3594613315782098
0.6781986014686510 0.8336776213852559 0.3491267247364505
0.5585922194781432 0.1117535948575537 0.3947061510630334
0.5816933405947111 0.5817426118472113 0.3911057142087202

Stable stishovite (100)

1.00000000000000
4.1772000000000000 0.0000000000000000 0.0000000000000000
0.0000000000000000 7.9953000000000003 0.0000000000000000
0.0000000000000000 0.0000000000000000 54.5315999999999974
Si O
39 78
Direct
0.4127212705138277 0.1571787758347156 0.3024563876770472
0.4127212705138277 0.4905127758347163 0.3024563876770472
0.4127212705138277 0.8238457758347124 0.3024563876770472
0.9131799999999970 0.3238399999999970 0.2631190000000032
0.9131799999999970 0.6571730000000002 0.2631190000000032
0.9131799999999970 0.9905070000000009 0.2631190000000032
0.8880728675921148 0.3237850785954350 0.4985410330723710
0.8880728675921148 0.6571190785954357 0.4985410330723710
0.8880728675921148 0.9904520785954318 0.4985410330723710
0.9123827964003084 0.3238371967215272 0.3417842640661064
0.9107290247845228 0.3237988327384826 0.4204103143792182
0.4118822277113381 0.1571408133238847 0.3810904426381470
0.4088223244300053 0.1571138747210412 0.4598458332794664
0.9123827964003084 0.6571701967215233 0.3417842640661064
0.9107290247845228 0.6571318327384859 0.4204103143792182
0.4118822277113381 0.4904748133238854 0.3810904426381470
0.4088223244300053 0.4904478747210419 0.4598458332794664
0.9123827964003084 0.9905041967215240 0.3417842640661064
0.9107290247845228 0.9904658327384865 0.4204103143792182
0.4118822277113381 0.8238078133238886 0.3810904426381470
0.4088223244300053 0.8237808747210451 0.4598458332794664
0.4136387294861734 0.8238352241652862 0.2237816123229521
0.4136387294861734 0.4905012241652855 0.2237816123229521
0.4136387294861734 0.1571682241652894 0.2237816123229521
0.9382871324078863 0.6572289214045668 0.0276969669276284
0.9382871324078863 0.3238949214045661 0.0276969669276284
0.9382871324078863 0.9905619214045700 0.0276969669276284
0.9139772035996927 0.6571768032784746 0.1844537359338929
0.9156309752154783 0.6572151672615121 0.1058276856207812
0.4144777722886630 0.8238731866761171 0.1451475573618524
0.4175376755699958 0.8239001252789606 0.0663921667205329
0.9139772035996927 0.3238438032784785 0.1844537359338929
0.9156309752154783 0.3238821672615160 0.1058276856207812
0.4144777722886630 0.4905391866761164 0.1451475573618524
0.4175376755699958 0.4905661252789599 0.0663921667205329
0.9139772035996927 0.9905098032784778 0.1844537359338929
0.9156309752154783 0.9905481672615153 0.1058276856207812
0.4144777722886630 0.1572061866761132 0.1451475573618524
0.4175376755699958 0.1572331252789567 0.0663921667205329

0.7698092578029119	0.1571291116032043	0.5154060070743611
0.7698092578029119	0.4904621116032004	0.5154060070743611
0.7698092578029119	0.8237951116032036	0.5154060070743611
0.2134881653599976	0.3238102254310178	0.3663341293047893
0.2123202283517784	0.3237907033131648	0.4450969649680943
0.6104679145762333	0.3238024038821976	0.3958138147281005
0.6197914312626125	0.3237859821711311	0.4740541858614975
0.7137175472560600	0.1571596263186947	0.3565383939315154
0.7117848450983644	0.1571277887470117	0.4351115084965613
0.1364254251409136	0.1571120824394328	0.4850894072614764
0.1103121789921389	0.1571314185151422	0.4056917271513854
0.2134881653599976	0.6571432254310210	0.3663341293047893
0.2123202283517784	0.6571237033131609	0.4450969649680943
0.6104679145762333	0.6571354038822008	0.3958138147281005
0.6197914312626125	0.6571189821711343	0.4740541858614975
0.7137175472560600	0.4904936263186954	0.3565383939315154
0.7117848450983644	0.4904617887470053	0.4351115084965613
0.1364254251409136	0.4904460824394334	0.4850894072614764
0.1103121789921389	0.4904654185151429	0.4056917271513854
0.2134881653599976	0.9904772254310217	0.3663341293047893
0.2123202283517784	0.9904577033131616	0.4450969649680943
0.6104679145762333	0.9904694038822015	0.3958138147281005
0.6197914312626125	0.9904529821711279	0.4740541858614975
0.7137175472560600	0.8238266263186986	0.3565383939315154
0.7117848450983644	0.8237947887470085	0.4351115084965613
0.1364254251409136	0.8237790824394295	0.4850894072614764
0.1103121789921389	0.8237984185151390	0.4056917271513854
0.1112030627303966	0.1571616438741614	0.3270446569816841
0.1112030627303966	0.4904956438741621	0.3270446569816841
0.1112030627303966	0.8238286438741653	0.3270446569816841
0.6113078630770019	0.3238329507927205	0.3172032841998771
0.6113078630770019	0.6571659507927237	0.3172032841998771
0.6113078630770019	0.9904999507927243	0.3172032841998771
0.2142425516874127	0.3238390842033141	0.2877011535545222
0.2142425516874127	0.6571720842033173	0.2877011535545222
0.2142425516874127	0.9905060842033180	0.2877011535545222
0.7143545951851920	0.1571729932017831	0.2778700986043887
0.7143545951851920	0.4905069932017838	0.2778700986043887
0.7143545951851920	0.8238399932017799	0.2778700986043887
0.0565507421967837	0.8238848883967975	0.0108319929256382
0.0565507421967837	0.4905518883968014	0.0108319929256382
0.0565507421967837	0.1572188883967982	0.0108319929256382
0.6128718346400035	0.6572037745689769	0.1599038706952101
0.6140397716482227	0.6572232966868370	0.0811410350319051
0.2158920854237678	0.6572115961177971	0.1304241852718988
0.2065685687373886	0.6572280178288707	0.0521838141385018
0.1126424527439411	0.8238543736813000	0.1696996060684839
0.1145751549016367	0.8238862112529901	0.0911264915034380
0.6899345748590804	0.8239019175605691	0.0411485927385229
0.7160478210078622	0.8238825814848596	0.1205462728486140
0.6128718346400035	0.3238707745689808	0.1599038706952101
0.6140397716482227	0.3238902966868409	0.0811410350319051
0.2158920854237678	0.3238785961178010	0.1304241852718988
0.2065685687373886	0.3238950178288675	0.0521838141385018
0.1126424527439411	0.4905203736813064	0.1696996060684839
0.1145751549016367	0.4905522112529894	0.0911264915034380
0.6899345748590804	0.4905679175605684	0.0411485927385229
0.7160478210078622	0.4905485814848589	0.1205462728486140
0.6128718346400035	0.9905367745689801	0.1599038706952101
0.6140397716482227	0.9905562966868402	0.0811410350319051
0.2158920854237678	0.9905445961178003	0.1304241852718988
0.2065685687373886	0.9905610178288669	0.0521838141385018
0.1126424527439411	0.1571873736813032	0.1696996060684839

```
 0.1145751549016367  0.1572192112529933  0.0911264915034380
 0.6899345748590804  0.1572349175605652  0.0411485927385229
 0.7160478210078622  0.1572155814848557  0.1205462728486140
 0.7151569372695974  0.8238523561258333  0.1991933430183153
 0.7151569372695974  0.4905183561258326  0.1991933430183153
 0.7151569372695974  0.1571853561258365  0.1991933430183153
 0.2150521369229992  0.6571810492072814  0.2090347158001293
 0.2150521369229992  0.3238480492072782  0.2090347158001293
 0.2150521369229992  0.9905140492072775  0.2090347158001293
 0.6121174483125884  0.6571749157966806  0.2385368464454771
 0.6121174483125884  0.3238419157966845  0.2385368464454771
 0.6121174483125884  0.9905079157966838  0.2385368464454771
 0.1120054048148091  0.8238410067982187  0.2483679013956106
 0.1120054048148091  0.4905070067982180  0.2483679013956106
 0.1120054048148091  0.1571740067982148  0.2483679013956106
```

Dense stishovite (110)

```
1.00000000000000
     7.9953000000000003    0.0000000000000000    0.0000000000000000
     0.0000000000000000    5.9074999999999998    0.0000000000000000
     0.0000000000000000    0.0000000000000000   36.1509000000000000
Si O
38 76
 Direct
 0.2340155388335880  0.8012375048130607  0.3916585970973121
 0.5666519209438117  0.8013094139785495  0.3916462363928219
 0.8992316078195304  0.8012774881684323  0.3916574397639300
 0.0666711868349918  0.3012471229790046  0.3916652529346372
 0.4007709435196672  0.3012803386168156  0.3916546333521786
 0.7324436594400267  0.3012780651798906  0.3916557597082093
 0.0666705889390223  0.8012521802263404  0.4749206494003927
 0.0666777227107470  0.8012232311789369  0.3083997043385424
 0.3998698826282219  0.8012157782669067  0.4754697498585100
 0.3998733462931918  0.8012094148641374  0.3078265650631940
 0.7333965737530731  0.8012257073757800  0.4754780329771310
 0.7333872943163119  0.8012293401090053  0.3078175302777971
 0.0666766792714128  0.3013589689897831  0.5518802918440570
 0.0666606223586652  0.3013297583314937  0.2314271270027132
 0.3996133628530593  0.3013300945368823  0.5598517797146426
 0.3995906890253872  0.3013024980234700  0.2234368910832495
 0.7337450083792985  0.3014290166591923  0.5598279576638177
 0.7337368614406858  0.3013933438569615  0.2234599487089560
 0.2363725758211653  0.3012349010787128  0.4738058821283191
 0.2363513849577772  0.3012234448435988  0.3095113143440826
 0.5666357823196124  0.3012481294307646  0.4758648856929278
 0.5666200659817922  0.3012531510073685  0.3074439200441040
 0.8969589381748007  0.3012028581810652  0.4737864815358118
 0.8969633049835433  0.3011892310380309  0.3095358124999815
 0.2251211918955054  0.8012639354026450  0.5593208608029538
 0.2250856814529059  0.8012400181777879  0.2239750937098017
 0.5666163525063387  0.8014159136178090  0.5593108140866794
 0.5666014517026999  0.8013794717136625  0.2239838954266818
 0.9080941462591136  0.8014132532908012  0.5592773645244302
 0.9081073864121197  0.8013882826084707  0.2240187816893759
 0.8703247807041981  0.3016387718545558  0.6382614973063582
 0.8702825427553409  0.3016203247143744  0.1450228326155805
 0.3733231282743661  0.8015924195272246  0.6422428154416298
 0.3733022206750641  0.8015616303062608  0.1410690588872596
 0.2629794183432766  0.3015723315512062  0.6382590662833136
 0.2629744149965424  0.3015315206142015  0.1450306728641615
 0.7596320798708935  0.8016609615058308  0.6422495168482637
```

0.7596151486573161	0.8016423786119785	0.1410664195543107
0.0666717372235506	0.3011933353256281	0.4420433000831125
0.0666678569751722	0.3011701291622416	0.3412865460556655
0.4022616131173410	0.3012399265272073	0.4426103880288634
0.4022236206950930	0.3012555163897632	0.3407006067972008
0.7310331388918090	0.3011966817674378	0.4425993725410654
0.7310436829150989	0.3012014497697367	0.3407116524448255
0.0666562939411178	0.8012646504753926	0.5245609583892005
0.0666463845047199	0.8012521238319507	0.2587421066975833
0.3970491992766697	0.8013062893402458	0.5281449509725874
0.3970411224309107	0.8012757029166004	0.2551574780293728
0.7362061793586639	0.8013522635473491	0.5281118009247071
0.7362031791853152	0.8013241860834214	0.2551936394571441
0.0666155938825785	0.3013203996049104	0.5048896332644314
0.0665930200521145	0.3012952803462660	0.2784231776413449
0.3987885165460628	0.3012292445165649	0.5071491587433862
0.3987620003869437	0.3012515411780907	0.2761570074610633
0.7345554196836451	0.3012017914843744	0.5071319220249548
0.7345551024912015	0.3011825525365665	0.2761755838573933
0.0664861330631032	0.8013177367571217	0.5900911621341217
0.0664748604801157	0.8013126679400288	0.1932034638975172
0.3925408365889296	0.8014312632875106	0.5942401282252532
0.3925166582507459	0.8013917931608564	0.1890705835970171
0.7403527496384509	0.8014663671001850	0.5942238945792894
0.7403463041789297	0.8014320748274952	0.1890863588271926
0.2344371134592304	0.9953290575198895	0.4746886507192512
0.2344281863356699	0.9953290856818171	0.3086197766112402
0.5665925871494388	0.9939749377688404	0.4758759439775622
0.5665764157026053	0.9939927877993873	0.3074286585502805
0.8988821069932790	0.9953240009181261	0.4746895839934243
0.8988862901810843	0.9953097908720763	0.3086236890576103
0.2277478754302267	0.4932131109571243	0.5578760375453469
0.2277096917421175	0.4931727554911540	0.2254086024859428
0.5666389307313880	0.4992893386714697	0.5579716012092381
0.5666332582617016	0.4992420362291475	0.2253125782702152
0.9056147631495204	0.4932847501434321	0.5578469187314381
0.9056183914110358	0.4932456318334805	0.2254325114912248
0.2344468446537661	0.6071624608253287	0.4746798809346556
0.2344344495964672	0.6071437597845949	0.3086267033792793
0.5665983411334017	0.6084583046375963	0.4758813721086516
0.5665945210735274	0.6084675012075639	0.3074242864907749
0.8988778167611918	0.6071633129020177	0.4746877997331208
0.8988852754074732	0.6071494596770551	0.3086238339389290
0.2277149645707110	0.1094674130652629	0.5578810192665640
0.2276854925854516	0.1094522226088109	0.2254044592196720
0.5666421513619144	0.1034521414197205	0.5579908217438562
0.5666323624004860	0.1034287881424251	0.2252932309989048
0.9055857990328565	0.1094851958850045	0.5578538749763048
0.9055976624682428	0.1094604866046812	0.2254328524306993
0.2874498753493076	0.0414984187716173	0.6572954380054392
0.2874916832238220	0.0414659028185129	0.1260045339654709
0.0666424568947751	0.3015068067034850	0.6243365435298616
0.0666160726282815	0.3014949509526849	0.1588995756774776
0.5664810248692224	0.8016798598124621	0.6560930602250301
0.5664595488068308	0.8016929387484569	0.1272563394464935
0.8457164589576640	0.0415191485303460	0.6572883487758454
0.8456347892833347	0.0414986073994170	0.1260106695866281
0.2874758038056749	0.5616355803479895	0.6572866470665333
0.2875375823163449	0.5615861286966594	0.1260140683702472
0.7343035768072943	0.3015090372210980	0.6063052757999225
0.7342683403927767	0.3014930848174641	0.1769840566344826
0.3992235160963756	0.3014682854118857	0.6063402978983090
0.3992340064276191	0.3014285411694464	0.1769490393645175

0.8456941963815595	0.5617347415479516	0.6572846387548353
0.8455673153671613	0.5617017292927362	0.1260032845061775
0.0665882438876359	0.8012054529028118	0.4238274367626804
0.0665944532727558	0.8012119373461013	0.3594871464617903
0.4007275955057224	0.8012532119836387	0.4239579720615889
0.4007044072672976	0.8012673549517414	0.3593499293680508
0.7325581658184817	0.8012089503253271	0.4239576496681415
0.7325731002669790	0.8012200507020518	0.3593497409197620
0.2341340710110987	0.1075215295771024	0.3916650795556769
0.5665975062421604	0.1074029050571743	0.3916487450002973
0.8991787790996750	0.1075388257399496	0.3916635079351916
0.2341109030050686	0.4949956618539096	0.3916586087812780
0.5665902987091278	0.4951358672732931	0.3916457320314822
0.8991812635281921	0.4949765677223325	0.3916599378401815
